# PCSK9 Inhibitors Reduce Oxidative Stress Biomarkers in Heterozygous Familial Hypercholesterolemia

**DOI:** 10.1111/jcmm.71206

**Published:** 2026-05-24

**Authors:** Agnieszka Woźniak‐Szczepocka, Agnieszka Pawlos, Paulina Gorzelak‐Pabiś, Marlena Broncel, Bożena Bukowska, Ewelina Woźniak

**Affiliations:** ^1^ Laboratory of Tissue Immunopharmacology, Department of Internal Diseases and Clinical Pharmacology Medical University of Lodz Lodz Poland; ^2^ Department of Biophysics of Environmental Pollution, Faculty of Biology and Environmental Protection University of Lodz Lodz Poland

**Keywords:** heterozygous familial hypercholesterolaemia, oxidative DNA damage, oxidative stress, PCSK9 inhibitors

## Abstract

*Proprotein convertase subtilisin/kexin type 9* (PCSK9) inhibitors, such as alirocumab and evolocumab, effectively reduce LDL‐C levels, improve cardiovascular outcomes, and are well tolerated in the treatment of heterozygous familial hypercholesterolemia (HeFH). Oxidative stress, increased in HeFH, leads to DNA damage, LDL oxidation (ox‐LDL), and reduced total plasma antioxidant capacity, which promotes the development of atherosclerosis. The aim of the study was to assess whether treatment with PCSK9 inhibitors reduces ox‐LDL, anti‐ox‐LDL antibodies, oxidative stress, and DNA damage, including 8‐OH‐Gua levels. The study included 40 patients with HeFH diagnosed clinically or genetically, and 33 healthy volunteers at low cardiovascular risk as controls. Blood samples were collected from all participants for lipid profile, Lp(a), oxidative stress markers (ox‐LDL, 8‐OHdG, anti‐oxLDL antibodies), total antioxidant capacity of plasma (TAC), and DNA damage status (using the comet assay with repair enzymes). The study showed that treatment with PCSK9 inhibitors (alirocumab or evolocumab) in HeFH patients significantly improved lipid profiles and reduced levels of oxidative stress markers such as 8‐OHdG and ox‐LDL, while increasing levels of anti‐ox‐LDL and TAC. This therapy also significantly reduced purine and pyrimidine DNA damage, although not to the level observed in the control group. The efficacy of reducing DNA damage was greater with alirocumab, which could be related to higher baseline levels of Lp(a) and oxidative damage in these patients. Treatment with PCSK9 inhibitors in HeFH patients reduces oxidative stress and DNA damage, indicating additional non‐lipid benefits that support their use in preventing atherosclerotic complications.

## 1 Introduction

Heterozygous familial hypercholesterolemia (HeFH) is one of the most common monogenic diseases, with an estimated global population prevalence of 1:200–1:300. In Poland, it occurs with a similar prevalence of approximately 1:250, representing approximately 150,000 individuals. Unfortunately, only about 5% of patients with HeFH are correctly diagnosed, and an even smaller percentage receive optimal treatment [1].

Proprotein convertase subtilisin/kexin type 9 inhibitors (PCSK9i) such as alirocumab and evolocumab are monoclonal antibodies that block PCSK9 interaction with LDL receptor (LDLR), increasing receptor availability on cell surfaces and enhancing LDL‐C clearance from plasma. These agents are well tolerated in HeFH, reducing LDL‐C by 50%–60% [2, 3]. Major trials (FOURIER and ODYSSEY OUTCOMES) demonstrated that alirocumab and evolocumab improve cardiovascular outcomes when added to statin therapy and show no safety differences compared to placebo [4, 5].

Oxidative stress is a significant factor in the development of cardiovascular disease (CVD), mainly through lipid peroxidation induced by reactive oxygen species (ROS). In endothelial cells (EC), especially in patients with familial hypercholesterolemia, the largest amounts of ROS come from dysfunction of the eNOS enzyme. The resulting ROS promote the oxidation of lipoproteins, especially LDL, forming ox‐LDL, which is associated with atherosclerosis and increased risk of ASCVD. OxLDL is actually an umbrella term reflecting a wide range of oxidative changes in the LDL particle, including the adduction of aldehydes such as malondialdehyde (MDA) to apolipoprotein B, the major protein of LDL [6]. B cells in the immune system can produce antibodies against various oxLDL epitopes (anti‐oxLDL). They are found in intimal lesions and circulating immune complexes, which confirms the relationship between atherosclerotic and thrombotic events. However, the role and significance of anti‐oxLDL antibodies is still poorly understood [7, 8]. High LDL levels are not only a substrate for ROS, but they themselves increase oxidative stress by reducing antioxidant levels and increasing the activity of ROS‐producing enzymes. In individuals with hypercholesterolemia, increased activity of these enzymes may lead to a decrease in plasma total antioxidant capacity (TAC). This may be due to a decrease in the activity of antioxidant enzymes or depletion of non‐enzymatic antioxidants [9]. Among the many types of DNA damage, ROS generated in familial hypercholesterolemia cause, among others, single‐strand breaks (SSB) and double‐strand breaks (DSB). In response to oxidative DNA damage, the activity of the base excision repair (BER) pathway increases. However, ox‐LDL lowers the level of enzymes in this pathway, including those responsible for the removal of 8‐oxoguanine (8‐OH‐Gua) [10]. The most common DNA damage associated with oxidative stress is 8‐OH‐Gua. In advanced atherosclerotic changes, its accumulation is observed in vascular smooth muscle cells, macrophages, and endothelial cells [11, 12]. Our previous work has shown that patients with HeFH have DNA damage, increased oxLDL and anti‐oxLDL levels, and lower total plasma antioxidant capacity compared to patients with normolipidemia [13]. In addition, in our previously published study, we demonstrated that PCSK9 inhibitors significantly reduced the level of DNA damage in the form of single‐ and double‐strand DNA breaks [14].

Therefore, the aim of this work is to determine whether PCSK9 inhibitors will affect the change in these parameters (ox‐LDL, anti‐ox‐LDL antibodies, total antioxidant capacity) and whether they affect the reduction of oxidative DNA damage to purines and pyrimidines and the reduction of 8‐OH‐Gua.

## 2 Materials and Methods

### 2.1 Patients With HeFH and Controls

The study group consisted of 40 patients who had been referred to the Lipid Disorders Treatment Center at the Department of Internal Medicine and Clinical Pharmacology in Bieganski Memorial Hospital, Lodz for hypercholesterolemia by their primary care physicians. All patients were referred from 01.04.2023 to 31.03.2024.

All patients had been diagnosed with primary familial hypercholesterolemia based on a score above eight points on the Dutch Lipid Clinic Network (DLCN) scale (assessing family history, clinical history, physical examination and LDL‐C level). Among the 40 patients, 15 were diagnosed with a genetic form of HeFH based on next‐generation sequencing (NGS) of FH‐related genes, that is, mutations in the LDL‐C receptor (LDLR), apolipoprotein B (APOB) or the proprotein subtilisin/kexin type 9 convertase gene (PCSK9) with confirmation by Sanger sequencing [15, 16].

The exclusion criteria comprised secondary causes of hypercholesterolemia, including hypothyroidism, kidney diseases, poorly controlled diabetes, cholestasis, or the use of drugs impairing lipid metabolism.

The control group consisted of 33 patients aged 27–64 years with low cardiovascular risk (SCORE2 < 2.5% for patients aged under 50 years, or < 5% for patients aged 50–69 years), free of medications, and no previous chronic or acute diseases in the past 3 months. In addition, no abnormalities were revealed under physical examination [16].

The investigation was approved by the Bioethics Committee of the Medical University of Lodz (RNN/10/23/KE). Informed consent was obtained from all participants. All methods were carried out in accordance with relevant guidelines and regulations.

### 2.2 Sample Collection and Diagnostic Laboratory Methods

The following data was collected from all participants during interviews, that is, cases and controls: personal history of hypertension, diabetes, smoking, cardiovascular disease, chronic kidney disease, non‐alcoholic fatty liver disease, pharmacological treatment, family history of hypercholesterolemia and cardiovascular disease. At the same time, all participants underwent a physical examination to determine the presence of corneal arcus and tendon xanthomas.

In both the control and research groups, peripheral blood mononuclear cells (PBMCs) and serum were isolated from peripheral whole blood. All blood samples were obtained after 10 h of fasting. PBMCs were isolated by a centrifugation series using a gradient medium for lymphocyte isolation. The isolated lymphocytes were suspended in a mix (45% RPMI medium, 45% bovine serum and 10% DMSO), then frozen at −80°C in a box with isopropanol and then stored in liquid nitrogen for up to 28 days.

The lipid profile, ALT, creatinine, eGFR, creatine kinase (CK) and Lp(a) levels were determined in patients before and after treatment. The lipid profile, consisting of total cholesterol (TCh), LDL‐C, triglycerides (TG), HDL‐C and non‐HDL levels, was then determined by colorimetric assay. All of the biochemical assays were performed using a Cobas 6000 or 8000 system (Roche, Switzerland). All serum or plasma samples intended for the sandwich ELISA analysis were aliquoted and stored at −80°C for later use. All biochemical measurements were performed in a central laboratory at Bieganski Hospital.

### 2.3 Enzyme‐Linked Immunosorbent Assay (ELISA)

The serum concentrations of 8‐OHdG were determined in the control and research groups by sandwich ELISA according to the manufacturer's instructions (The Japan Institute for the Control of Aging JaICA, Japan). The serum concentrations of ox‐LDL and anti‐ox‐LDL and the level of plasma antioxidant capacity in the control and study groups were also determined by the sandwich ELISA method according to the manufacturer's instructions (Immunodiagnostik AG, Germany).

### 2.4 Comet Assay

#### 2.4.1 Alkaline Version With DNA Repair Enzyme Treatment (Purine and Pyrimidine Detection)

Damages of purines and pyrimidines were assessed by modification of the comet assay (by single cell gel electrophoresis) with repair enzymes. The comet assay was conducted according to Woźniak et al. [17]. Detection of oxidative DNA damage was conducted using bifunctional endonuclease III (Nth) and formamidopirymidyne DNA glycosylase (FPG) (BioLabs, USA).

#### 2.4.2 Comet Analysis

The comets were observed at 200× magnification under a fluorescence microscope (Zeiss Axio Scope.A1) connected to an Axiocam 305 colour video camera (Carl Zeiss AG, Oberkochen, Germany). The microscope was connected to a desktop PC equipped with Lucia‐Comet v. 7.60 software (Laboratory Imaging, Praha, Czech Republic). One hundred images (comets) were randomly selected from each sample and the mean value of DNA in the comet tail was taken as an index of DNA damage (expressed in percent).

### 2.5 Statistical Analyses

The normality of the distribution of the acquired data was checked using the Shapiro–Wilk test. Some data demonstrated a normal distribution, and parametric tests were used, while others were not normally distributed and were tested with non‐parametric tests; the choice of test is given in the description of each figure. Statistical analyses were performed with GraphPad Prism 9.0 (GraphPad Software, San Diego, California, United States). All tests were considered significant at a *p*‐value below 0.05.

## 3 Results

The study group (*n* = 40 patients) consisted of HeFH patients treated with the PCSK9 inhibitors evolocumab and alirocumab. Of these participants, 15 (37.5%) were men, and 25 (62.5%) were women. The median age was 59 years (IQR, 28–82 years). The control group consisted of 33 patients with low cardiovascular risk. This group was significantly younger than the study group (*p* = 0.010); however, no significant correlations were found between age and the studied parameters. No significant difference in sex dominance was observed between groups (*p* > 0.05).

In the study group, the following parameters were analyzed before treatment with iPCSK9 and again after 6 months of treatment (Table S1): lipid profile, Lp(a), creatinine, eGFR, creatine kinase (CK), ALT, hsCRP, FT4, TSH, oxidative damage to purines and pyrimidines and the main parameters responsible for oxidative balance such as the level of the 8‐hydroxy‐2‐deoxyguanosine (8‐OHdG), oxidized‐LDL (ox‐LDL), anti‐oxidized‐LDL (anti‐ox‐LDL) and total antioxidative capacity (TAC). The creatinine, eGFR, creatine kinase (CK), ALT, hsCRP, FT4 and TSH levels remained normal throughout the study period and did not change from baseline.

The clinical data, including sex, age, pre‐existing medical conditions, FH diagnosis, characteristic features and lipid‐lowering treatment, are given in Table 1.

**TABLE 1:** Characteristics of the subjects with controls and HeFH.

Parameter median/(IQR)	Controls, *N* = 33	Patients with HeFH, *N* = 40
Sex, *n* (%)		
Female	28 (84.8%)	25 (62.5%)
Male	5 (15.2%)	15 (37.5%)
Age (years)	48 (27; 64)	59 (28; 82)
Cardiovascular diseases		
Acute coronary syndrome	0 (0%)	9 (22.5%)
Chronic coronary syndrome	0 (0%)	19 (47.5%)
Lower extremity arterial disease (LEAD)	0 (0%)	5 (12.5%)
Carotid Atherosclerosis (CAS)	0 (0%)	26 (65%)
Stroke	0 (0%)	3 (7.5%)
Myocardial infarction	0 (0%)	6 (15%)
Hypertension	0 (0%)	24 (60%)
Diabetes mellitus	0 (0%)	4 (10%)
Smoking	0 (0%)	6 (15%)
Chronic kidney disease	0 (0%)	4 (10%)
Non‐alcoholic fatty liver disease	0 (0%)	3 (7.5%)
DLCN score	0 (0%)	40 (100%)
Patients genotype		
LDLR mutation	0 (0%)	14 (35%)
ApoB mutation	0 (0%)	1 (2.5%)
PCSK9 mutation	0 (0%)	0 (0%)
Corneal arcus	0 (0%)	10 (25%)
Tendon Xanthomas	0 (0%)	11 (27.5%)
Xantelasma	0 (0%)	2 (5%)
Alirocumab	0 (0%)	22 (55%)
Evolokumab	0 (0%)	18 (45%)

### 3.1 HeFH Patients Treated With PCSK9i Have Lower DNA Damage to the Purine and Pyrimidines Was Observed Than Before Treatment

Significantly higher oxidative damage of purines and pyrimidines was noted in the HeFH patients (27.02 [20.86–33.55] % DNA damage purines; 21.85 [17.95–28.31] % DNA damage pyrimidines) compared to controls (8.53 [6.54–10.77] % DNA damage purines; 7.76 [5.64–8.99] % DNA damage pyrimidines).

PCSK9i treatment resulted in lower levels of nitrogenous bases damage: purines (21.72 [15.08–26.83] % DNA damage in the comet tail) and pyrimidines (20.56 [15.36–25.48] % DNA damage in the comet tail). The differences between the groups were statistically significant (*p* < 0.001; *p* < 0.01).

**FIGURE 1:**
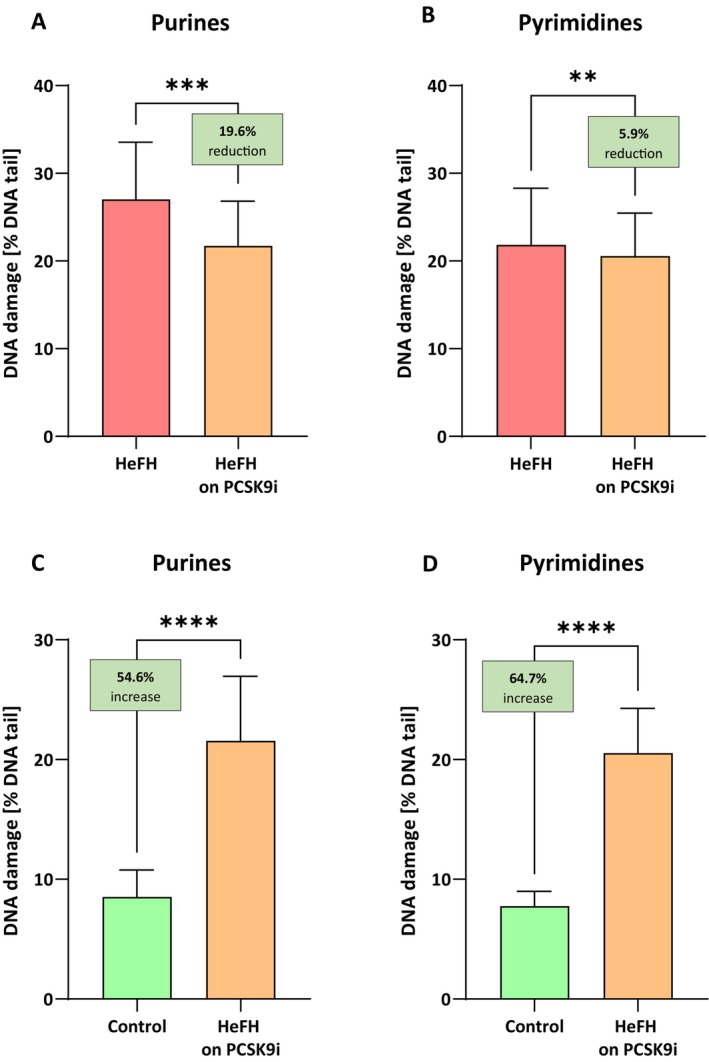
(A, B) Percentage of DNA damage in the comet tail for damage purines and pyrimidines of patients with HeFH before and after treatment PCSK9i. For controls % DNA damage in comet tail is: For purines (8.53 [6.54–10.77]) (A), for pyrimidines (7.76 [5.64–8.99]) (B). (C, D) Percentage of DNA in the comet tail for damage purines (C) and pyrimidines (D) of controls and patients with HeFH after treatment PCSK9i. In box shown % reduction/increase as a median. Significant differences are indicted by ****p < 0.0001; ***p < 0.001, **p < 0.01. Statistical analysis was conducted using the Wilcoxon test (A, B) and the Mann–Whitney test (C, D).

The percentages of DNA in the comet tail for purines and pyrimidines of the HeFH patients, before and after PCSK9i treatment, are shown in Figure 1A,B. Although PCSK9i therapy significantly reduced DNA damage, it did not fall to that noted in the controls: for purines (8.53 [6.54–10.77] versus 21.56 [17.84–26.53] % DNA damage in the comet tail (*p* < 0.0001)) and for pyrimidines (7.76 [5.64–8.99] versus 20.54 [15.98–24.27] % DNA damage in the comet tail (*p* < 0.0001)) (Figure 1C,D).

### 3.2 Both Alirocumab and Evolocumab Reduce Levels of DNA Damage to the Purine and Pyrimidines

**FIGURE 2:**
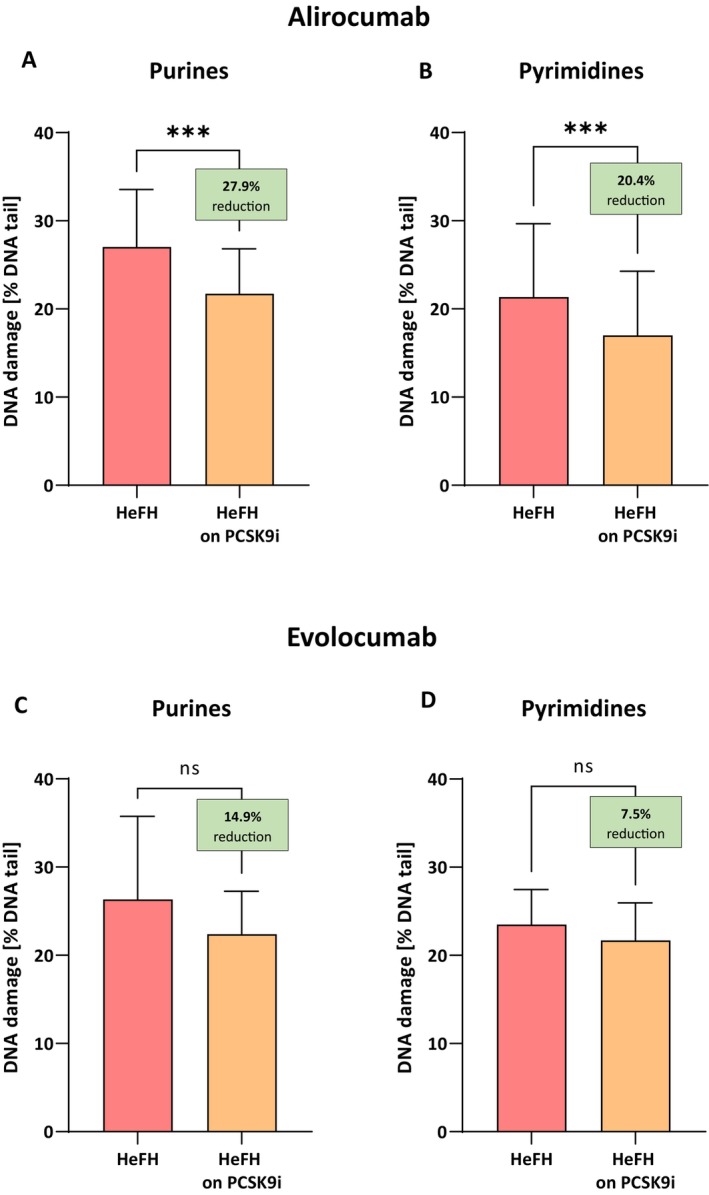
Percentage of DNA damage in the comet tail from damage purines and pyrimidines of patients with HeFH before and after treatment alirocumab/evolocumab. In boxes shown % reduction as a median. Significant differences are indicated by ***p < 0.001—statistical analysis was conducted using the Wilcoxon test. No significant differences are indicated by *p* = 0.0775; *p* = 0.1893—statistical analysis was performed using paired Student's *t* test.

Alirocumab treatment was associated with a significant reduction in DNA damage for purines, that is, 19.67 [12.42–25.75] % DNA damage in comet tail, compared to before treatment, that is, 27.29 [24.55–33.25] % DNA damage in comet tail (this difference was statistically significant *p* < 0.001). For pyrimidines a significant reduction in DNA damage was also observed, that is, 17.00 [11.93–24.26] % DNA damage in comet tail, compared to before treatment, that is, 21.36 [16.00–29.67] % DNA damage in comet tail (this difference was statistically significant *p* < 0.001) (Figure 2A,B).

In the case of evolocumab, we can observe a trend of reducing damage at the level of 14.9% for purine damage (22.39 [17.65–27.25] vs. 26.33 [18.82–35.76] % DNA damage in comet tail) with a significance level of *p* = 0.0775 and 7.5% for pyrimidine damage (21.71 [18.02–25.97] vs. 23.48 [18.72–27.47] % DNA damage in comet tail) with a significance level of *p* = 0.1893; however, this reduction is not statistically significant (Figure 2C,D).

### 3.3 Patients Treated With Alirocumab Had Higher Baseline Oxidative Damage to Purines and Pyrimidines

Baseline purine damage values for alirocumab‐treated patients were 27.29 [24.55–33.25] % DNA damage, compared to evolocumab‐treated patients 21.46 [18.32–26.77] % DNA damage in the comet tail (*p* < 0.05).

Baseline pyrimidine damage values for alirocumab‐treated patients were 23.44 [19.00–34.52] % DNA damage, compared to evolocumab 21.76 [18.26–25.25] % DNA damage in the comet tail (*p* = 0.2625).

**FIGURE 3:**
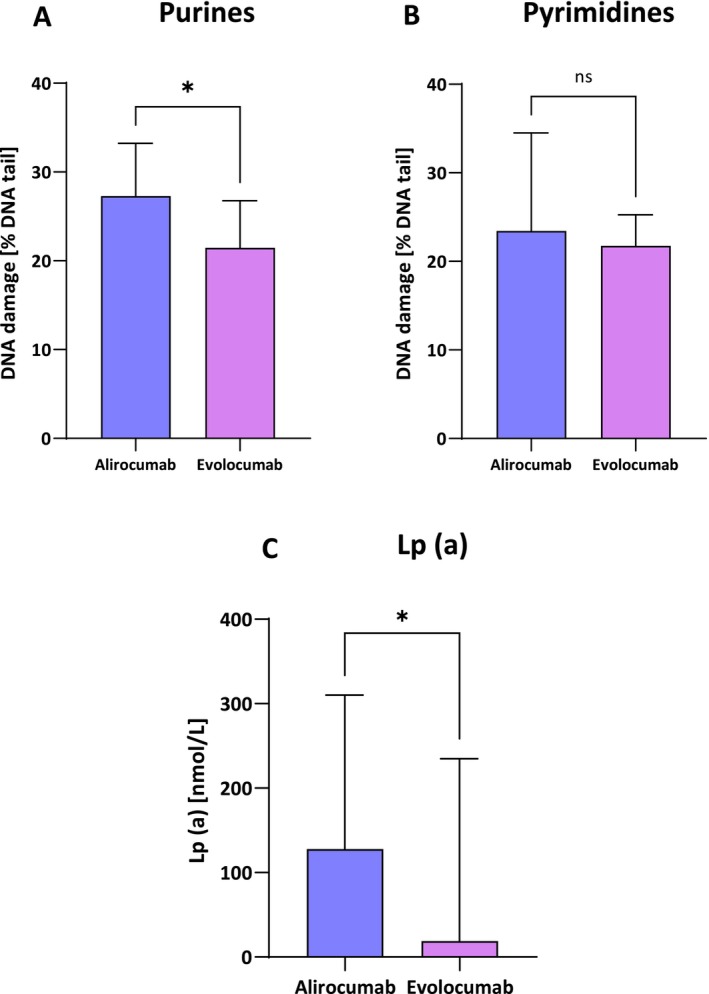
Percentage of DNA damage in the comet tail caused by purine and pyrimidine damage in HeFH patients before treatment with alirocumab (A) and evolocumab (B). Baseline lipoprotein (a) levels in patients treated with alirocumab/evolocumab (C). Significant differences are indicated by *p < 0.05—statistical analysis was performed using the Mann–Whitney test (C) and the unpaired Student's *t* test (A). No significant differences are indicated by *p* = 2625—statistical analysis was performed using the paired Mann–Whitney test (B).

In our previous study we observed a correlation between DNA damage and Lp(a) levels, and this time we confirmed that alirocumab‐treated patients with baseline, higher values of oxidative damage to purines and pyrimidines had higher baseline Lp(a) values 127.9 [81.15–31.03] nmol/L vs. evolocumab 18.9 [6.15–234.8] nmol/L (*p* < 0.05). These dependencies are shown in Figure 3.

### 3.4 Serum Levels of 8‐OHdG Protein Are Significantly Lower in Patients After Treatment With PCSK9 Inhibitors

**FIGURE 4:**
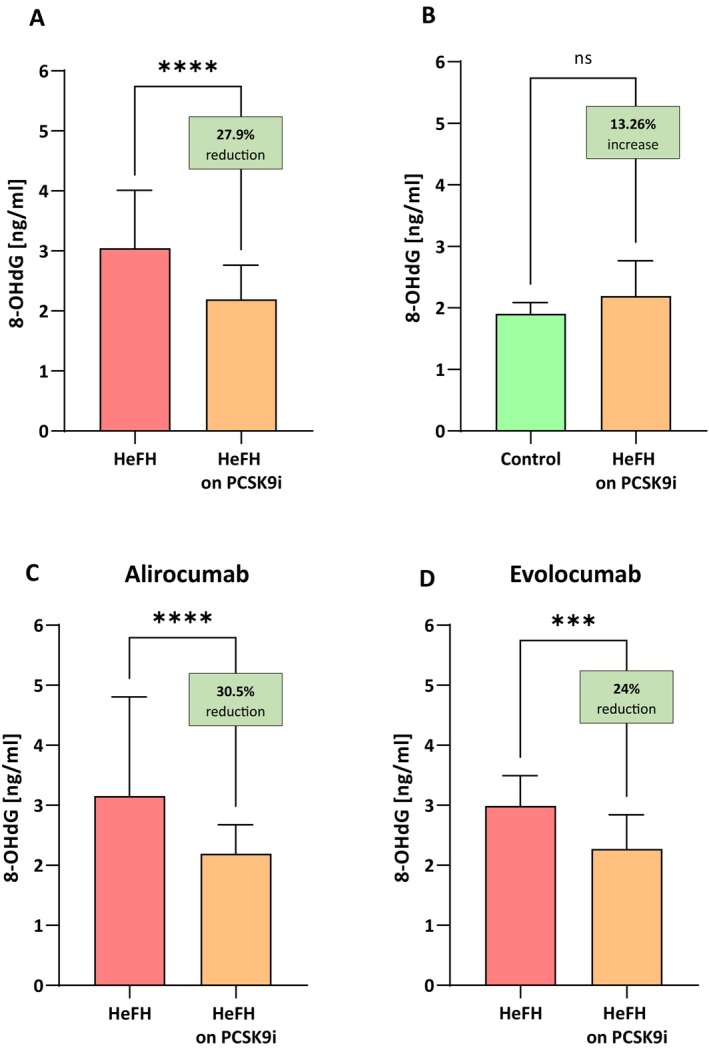
(A) Serum levels of 8‐OHdG in patients with HeFH before and after treatment with PCSK9 inhibitors. (B) Serum levels of 8‐OHdG in controls and patients with HeFH after treatment with PCSK9 inhibitors. (C, D) 8‐OHdG level of individual patients before and after treatment alirocumab/evolocumab. In box shown % reduction as a median. Significant differences are indicted by ****p < 0.0001; ***p < 0.001. Statistical analysis was conducted using the Wilcoxon test (A, C), Mann–Whitney test (B) and the paired student *t*‐test (D).

The HeFH patients had significantly lower levels of 8‐OHdG after treatment (evolocumab or alirocumab), that is, 2.194 [1.668–2.765] ng/ml than before treatment, that is, 3.044 [2.700–4.013] ng/mL, (*p* < 0.0001). No significant differences in 8‐OHdG reduction were observed between alirocumab and evolocumab (*p* = 0.8878). The serum levels of 8‐OHdG in patients with HeFH before and after treatment are shown in Figure 4A,C,D.

PCSK9i therapy was found to reduce 8‐OHdG to a level lower than in the normolipidemic group: 1.903 [1.731–2.088] ng/mL versus 2.194 [1.668–2.765] ng/mL (*p* = 0.052; Figure 4B).

### 3.5 Serum Levels of Ox‐LDL Are Significantly Lower in Patients After Treatment With PCSK9 Inhibitors

**FIGURE 5:**
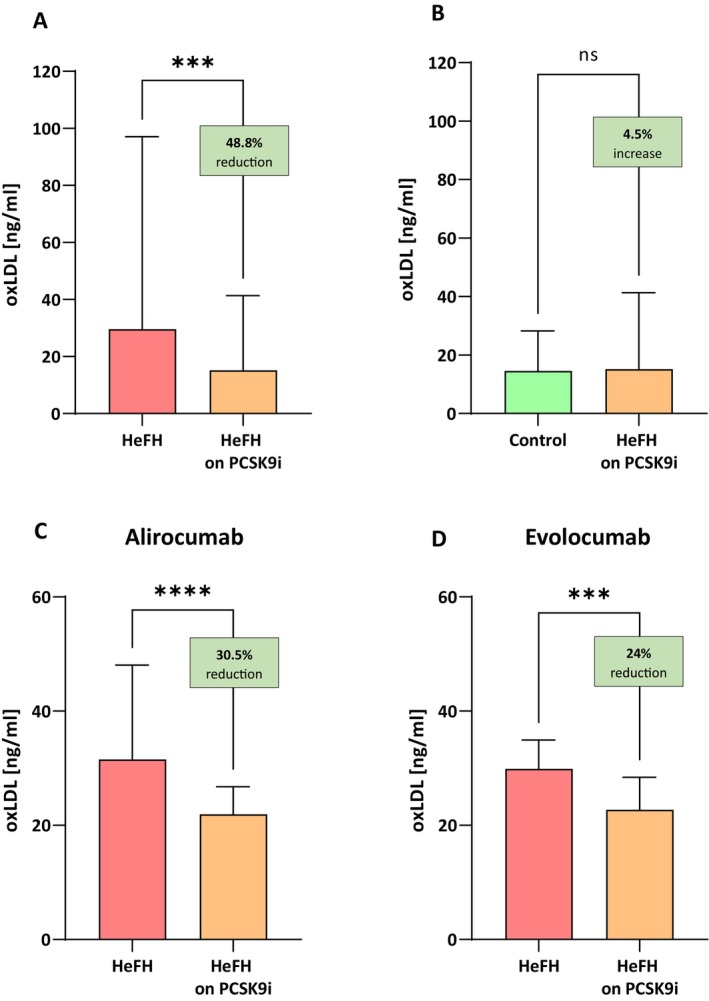
(A) Serum levels of ox‐LDL in patients with HeFH before and after treatment with PCSK9 inhibitors. (B) Serum levels of ox‐LDL in controls and patients with HeFH after treatment with PCSK9 inhibitors. (C, D) Ox‐LDL level of individual patients before and after treatment alirocumab/evolocumab. In box shown % reduction/increase as a median. Significant differences are indicted by ****p < 0.0001; ***p < 0.001. Statistical analysis was conducted using the Wilcoxon test (A, C), Mann–Whitney test (B) and the paired student *t*‐test (D).

PCSK9i treatment resulted in lower levels of ox‐LDL after treatment, that is, 15.18 [6.46–41.34] ng/mL vs. 29.65 [10.61–97.12] ng/mL, (*p* < 0.001). No significant differences in ox‐LDL reduction were observed between alirocumab and evolocumab (*p* = 0.8878). The serum levels of ox‐LDL in patients with HeFH before and after treatment are shown in Figure 5A,C,D.

PCSK9i therapy was found to reduce ox‐LDL to a level lower than in the control group: 15.18 [6.46–41.34] ng/mL versus 14.53 [3.81–28.27] ng/mL (*p* = 0.2047; Figure 5B).

### 3.6 Serum Levels of Antiox‐LDL Are Significantly Higher in Patients After Treatment With PCSK9 Inhibitors

**FIGURE 6:**
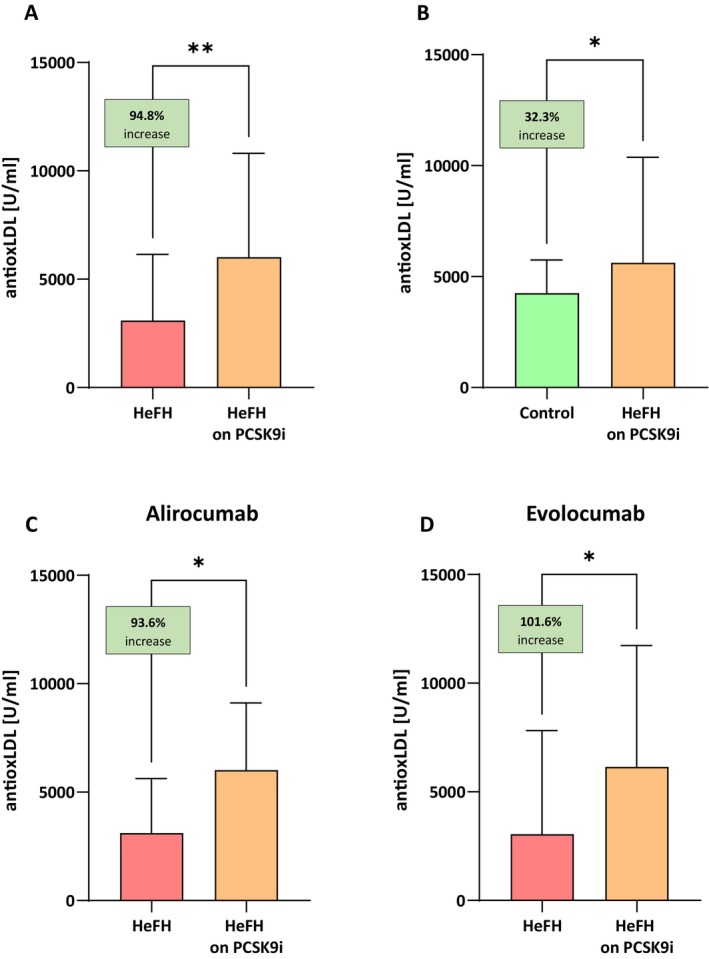
(A) Serum levels of antiox‐LDL in patients with HeFH before and after treatment with PCSK9 inhibitors. (B) Serum levels of antiox‐LDL in controls and patients with HeFH after treatment with PCSK9 inhibitors. (C, D) Antiox‐LDL level of individual patients before and after treatment alirocumab/evolocumab. In box shown % increase as a median. Significant differences are indicated by **p < 0.01, *p < 0.05. Statistical analysis was conducted using the Wilcoxon test (A, C, D) and Mann–Whitney test (B).

The HeFH patients had significantly higher levels of antiox‐LDL after treatment (alirocumab/evolocumab), that is, 6020 [3765–10,805] U/mL than before treatment, that is, 3090 [1448–6143] U/mL, (*p* < 0.01). No significant differences in antiox‐LDL reduction were observed between alirocumab and evolocumab (*p* = 0.6621). The serum levels of antiox‐LDL in patients with HeFH before and after treatment are shown in Figure 6A,C,D.

PCSK9i therapy was found to increase antiox‐LDL to a higher level than in the control group: 5625 [3340–10,375] U/mL versus 4250 [2478–5753] U/mL (*p* < 0.05; Figure 6B).

### 3.7 Serum Levels of TAC Are Significantly Higher in Patients After Treatment With PCSK9 Inhibitors

**FIGURE 7:**
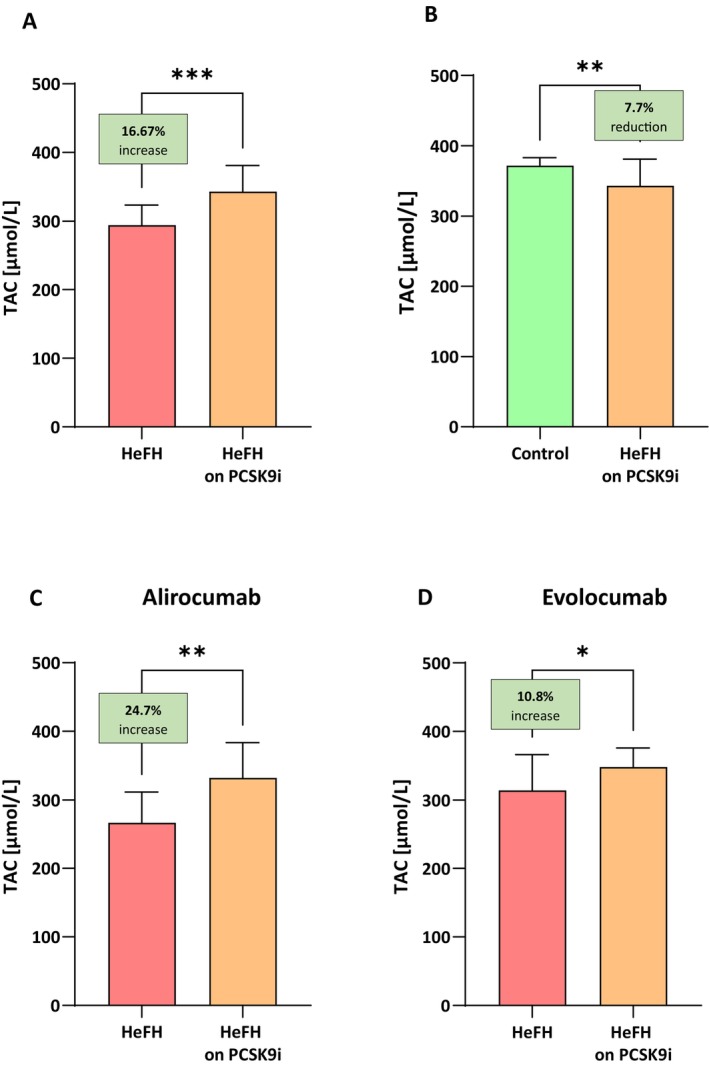
(A) Levels of total antioxidant capacity of plasma in patients with HeFH before and after treatment with PCSK9 inhibitors. (B) Levels of total antioxidant capacity of plasma in controls and patients with HeFH after treatment with PCSK9 inhibitors. (C, D) Levels of total antioxidant capacity of plasma for individual patients before and after treatment alirocumab/evolocumab. In box shown % increase/reduction as a median. Significant differences are indicted by ***p < 0.001, **p < 0.01, *p < 0.05. Statistical analysis was conducted using the Wilcoxon test (A, C), Mann–Whitney test (B) and the paired student *t*‐test (D).

The HeFH patients had significantly higher levels of TAC after treatment (evolocumab or alirocumab), that is, 342.9 [286.9–380.9] μmol/L than before treatment, that is, 293.9 [246.4–323.5] μmol/L/mL, (*p* < 0.001). The TAC parameter in patients with HeFH before and after treatment are shown in Figure 7A,C,D.

PCSK9i therapy was found to increase TAC in HeFH patients but not to the levels of the control group: 342.9 [286.9–380.9] μmol/L versus 371.6 [357.6–383.1] μmol/L/mL (*p* = 0.0061; Figure 7B).

## 4 Discussion

We have found in previous studies that PCSK9 inhibitor therapy reduces the level of single‐ and double‐stranded DNA damage in HeFH patients, regardless of the type of inhibitor used. This is not due to the effect of lowering LDL cholesterol or Lp(a) levels, but is most likely due to the pleiotropic properties of iPCSK9 (Figure S1). In the present study, we have shown that oxidative DNA damages to purines and pyrimidines is also reduced in HeFH patients after treatment with PCSK9 inhibitors. To further characterize the determinants of oxidative DNA damage, we additionally performed a multiple linear regression analysis including both HeFH patients and control subjects. In this model, oxidative DNA damage was used as the dependent variable, while age and FH status were included as independent variables. The analysis demonstrated that FH status was the major determinant of oxidative DNA damage (*β* = 18.91; *p* < 0.0001), whereas age was not significantly associated with DNA damage (*β* = 0.122; *p* = 0.142) (Table S2). Available evidence suggests that evolocumab may have more potent antioxidant effects than alirocumab in vivo in patients at high cardiovascular risk, particularly in the context of endothelial cell protection and reduction of oxidative stress associated with atherosclerosis [18, 19]. There is still a lack of other literature data on the level of DNA damage, both regarding single‐ and double‐stranded breaks and oxidative damage within nitrogenous bases.

Alirocumab is much more effective in reducing oxidative damage to both purines and pyrimidines. In the case of evolocumab, we only observe a trend of reducing oxidative damage to both purines and pyrimidines. However, it should be emphasized that the comparison between patients treated with alirocumab and evolocumab is based on relatively small subgroups, and therefore increasing the sample size is necessary to reliably assess potential differences between the two treatments. The difference in the effectiveness of reducing oxidative damage between inhibitors may result from the initial differences in the levels of oxidative damage, which were higher in patients assigned to alirocumab treatment than in patients assigned to evolocumab treatment. In turn, the higher level of oxidative damage in patients assigned to alirocumab treatment can be explained by the initial higher level of Lp(a) in these patients, which is consistent with our previous analyses, which showed that the level of DNA damage increases with the level of Lp(a). There are literature data proposed by the group of researchers Watts F.G. et, who in their studies showed that alirocumab achieves the greatest effectiveness in patients with very high levels of Lp(a), especially in combination with statins. Its effect on apo(a) production is revealed only at extreme levels of this lipoprotein. Evolocumab, on the other hand, has a stronger effect in monotherapy. In combination with atorvastatin, its effect on apo(a)/Lp(a) production is weakened, although catabolism remains elevated [20, 21]. Importantly, the results of the recent VESALIUS‐CV trial confirm the clinical relevance of PCSK9 inhibition. In this randomized, placebo‐controlled study, evolocumab significantly reduced the risk of first major cardiovascular events (MACE) in high‐risk patients with diabetes but without confirmed significant atherosclerosis. These findings suggest that the benefits of PCSK9 inhibitors may extend beyond patients with advanced atherosclerosis and could be relevant already at earlier stages of cardiovascular risk assessment. These studies also confirm the necessity and validity of expanding research on the pleiotropic properties of these inhibitors Moreover, further studies in larger cohorts are needed to better elucidate the differential effects of individual PCSK9 inhibitors [22, 23]. Besides monoclonal PCSK9 inhibitors, another available lipid‐lowering therapy targeting the PCSK9 pathway is inclisiran. It is a small interfering RNA that reduces hepatic synthesis of PCSK9. Inclisiran plays an important therapeutic role alongside the described monoclonal antibodies in patients with HeFH and is supported by current guidelines and expert consensus [24]. However, as inclisiran was not evaluated in the present study, its potential pleiotropic and antioxidant effects remain insufficiently characterized. Therefore, further studies are needed to assess whether inclisiran exerts similar effects on oxidative stress and DNA damage as monoclonal PCSK9 inhibitors.

PCSK9 inhibitors, in addition to their effect on LDL reduction, in the range of 50%–60% when used as an adjunct to statin ± ezetimibe therapy, directly affect the level of oxidative stress markers, such as ferric reducing antioxidant power (FRAP), reduction of hydroperoxides and malondialdehyde (MDA) levels or increased cell viability in response to H_2_O_2_ [25]. In the literature, additional evidence has been presented supporting the pleiotropic effects of PCSK9 inhibitors. Punch et al. highlighted that these agents may exert beneficial effects not only through lipid lowering, but also by reducing oxidative stress, inflammatory responses, and processes involved in atherogenesis. To support the role of PCSK9 in inflammatory activation, the authors pointed to the structural homology between the C‐terminal CRD of PCSK9 and the resistin domain, which is associated with pro‐inflammatory stimulation [26].

Similarly, Vlachopoulos et al. demonstrated that long‐term use of PCSK9 inhibitors is associated with reduced vascular inflammatory activity, as reflected by decreased FDG uptake in vascular imaging studies. These findings may suggest plaque stabilization and a reduction of inflammatory activity within the arterial wall [27]. Furthermore, in vivo models have shown that evolocumab treatment reduced the level of reactive oxygen species in the aorta and reduced the level of the oxidative damage marker 8‐hydroxy‐2′‐deoxyguanosine (8‐OHdG) [19]. Increased levels of 8‐hydroxy‐2′‐deoxyguanosine (8‐OHdG) are significantly associated with the occurrence and severity of cardiovascular disease (CVD). A meta‐analysis of over 1800 participants showed that patients with CVD have significantly higher levels of 8‐OHdG in blood and urine compared to healthy individuals. Additionally, higher levels of 8‐OHdG correlate with the severity of atherosclerosis and the number of coronary artery involvement [28, 29]. However, there are no data from clinical studies on the effect of PCSK9 inhibitors (alirocumab and evolocumab) on 8‐OHdG levels in patients with HeFH.

Patients after treatment with PCSK9 inhibitors achieve lower values of modified guanine, but these values are not at the level of the control group. Moreover, both alirocumab and evolocumab significantly reduced the level of the 8‐OHdG marker and no differences were found in the inhibitor used. There is evidence that a decrease in 8‐OHdG may indicate the activity of repair enzymes (e.g., OGG1), which effectively remove damage but this requires further research [30].

Both alirocumab and evolocumab treatment significantly reduced serum circulating ox‐LDL levels, and there was no difference in the inhibitor used. These results are consistent with those obtained by Cammisotto et al., who reported that after 6 months of treatment with PCSK9 inhibitors, ox‐LDL levels decreased by about 40% (in our study by 48%) [31].

In patients with very high cardiovascular risk, such as HeFH patients, vessel walls with a high ox‐LDL burden may be at higher risk of future atherosclerotic plaque formation and rupture. Therefore, ox‐LDL is an important marker of ‘vulnerable plaque’, but also provides information on how targeted therapy opens up new treatment options [32, 33]. Interestingly, the results of the study conducted by Lankin et al. indicate that PCSK9 inhibitors, in addition to lowering cholesterol levels, also significantly reduce atherogenic oxidized LDL and do not affect the activity of key antioxidant enzymes, which, in the absence of detected adverse effects, is very promising in the treatment of hypercholesterolemia. As Lankin et al. prove, the reduction in the level of ox‐LDL in plasma is not just a simple reflection of the decrease in the number of LDL particles, but may indicate the activation of unknown mechanisms regulating free radical oxidation under the influence of the PCSK9 inhibitor [34].

Patients with HeFH have a high rate of cardiovascular disease, including coronary artery disease and heart failure. Reduced anti‐oxLDL antibodies have been reported in both coronary artery disease and heart failure, but the data in the literature are currently inconsistent [6, 35]. Our results show a statistically significant increase in anti‐oxLDL antibodies in patients treated with PCSK9 inhibitors, moreover above the level of the control group. No significant differences were observed between alirocumab and evolocumab treatment. However, there are no studies on the effect of PCSK9 inhibitors on anti‐oxLDL antibody levels in patients with HeFH. J van den Berg et al. proposed a review of available studies on the association of anti‐oxLDL antibodies with improvement of cardiovascular parameters, suggesting an association between anti‐oxLDL antibodies and coronary artery disease, especially in the case of the IgM subclass [6]. Although the available results are ambiguous, statin therapy did not have a significant effect on the level of IgG and IgM antibodies to oxLDL [36], which supports the efficacy of PCSK9 inhibitors. Anti‐oxLDL may play a role in modifying atherogenesis; it has not been definitively established whether autoantibodies against OxLDL have an independent predictive value as biomarkers beyond traditional cardiovascular risk factors.

The induction of autophagy in endothelial cell macrophages is closely related to the antioxidant capacity of the organism, because autophagy is one of the key mechanisms limiting oxidative stress at the cellular level [19]. Total antioxidant capacity (TAC) of plasma indicates the cumulative activity of all antioxidant groups [37]. Treatment with PCSK9 inhibitors such as alirocumab and evolocumab increases the level of TAC. No significant differences between the inhibitors used were observed. In the study by Ganjali et al., higher levels of thiols and prooxidant‐antioxidant balance (PAB) were also observed compared to the control group. The level of antioxidant enzymes was also assessed in this work. There were no significant differences in the activity of antioxidant enzymes (GPx, SOD, CAT) in patients with HeFH and patients with HoFH, which may indicate the need for intensified treatment with PCSK9 inhibitors [38]. It is suggested that PCSK9 inhibitors may affect the SIRT3 protein, a mitochondrial deacetylase that has the ability to increase the activity of antioxidant enzymes, while their effect on pro‐antioxidant parameters requires further research [39].

## 5 Limitations

This study has some limitations. The first is the small size of the control and study groups, which may be attributed to the inclusion of only patients with low cardiovascular risk in the control group and the low percentage of correctly diagnosed patients in Poland (5%) in the study group. However, this work is intended as a pilot for future studies with larger and more diverse cohorts, and including a wider range of PCSK9i lipid‐lowering treatments.

Another limitation of the present study is the age difference between the control and study groups. Although we did not observe a correlation between age and the investigated parameters, it should be emphasized that age is a well‐established factor affecting both DNA damage and cardiovascular risk. To evaluate the potential confounding effect of age, we performed a multivariable linear regression analysis specifically for this purpose, including age as a covariate in a model assessing oxidative DNA damage. In this analysis, age was not a statistically significant predictor of oxidative DNA damage (*p* > 0.05). Moreover, although the HeFH group is significantly older than the healthy group, the effect of familial hypercholesterolaemia on oxidative DNA damage remains significant in the multiple linear regression model after adjustment for age. Familial hypercholesterolaemia is an age‐independent risk factor for oxidative DNA damage.

## 6 Conclusions

Treatment with PCSK9 inhibitors in HeFH patients reduces oxidative stress and DNA damage, indicating additional non‐lipid benefits that support their use in preventing atherosclerotic complications.

## Author Contributions

**Paulina Gorzelak‐Pabiś:** investigation, resources, writing – review and editing, supervision. **Agnieszka Woźniak‐Szczepocka:** conceptualization, methodology, formal analysis, investigation, visualization, writing – original draft, writing – review and editing, project administration, funding acquisition. **Agnieszka Pawlos:** resources. **Marlena Broncel:** investigation, supervision, writing – review and editing. **Ewelina Woźniak:** conceptualization, methodology, formal analysis, writing – original draft, writing – review and editing, supervision, investigation. **Bożena Bukowska:** methodology.

## Funding

The research was financed by the National Science Center as part of the ‘Preludium‐21’ research project, UMO‐2022/45/N/NZ7/01622.

## Conflicts of Interest

Agnieszka Wozniak reports financial support was provided by National Science Centre Poland. Agnieszka Wozniak reports a relationship with National Science Centre Poland that includes: funding grants. If there are other authors, they declare that they have no known competing financial interests or personal relationships that could have appeared to influence the work reported in this paper.

## Supporting information


**Figure S1:** The heatmap Spearman's rank correlation with age for the study groups: (A) patients with HeFH before treatment with PCSK9 inhibitors, (B) HeFH patients after treatment with PCSK9 inhibitors, and (C) the control group. Blue squares indicate significant positive correlations (*r* > 0.5, *p* < 0.05), white squares indicate non‐significant correlations (*p* > 0.05) and red squares indicate significant negative correlations (*r* < −0.5, *p* < 0.05).

**Table S1:** Serum levels of lipid parameters in controls and HeFH patients before and after treatment with PCSK9 inhibitors (Supporting Information).

**Table S2:** Multivariable linear regression model evaluating the effect of familial hypercholesterolemia (HeFH) on oxidative DNA damage with age included as a covariate.

## Data Availability

Data openly available in a public repository that issues datasets with DOIs. Anonymized data supporting the main findings of this study are available in the Zenodo repository (https://doi.org/10.5281/zenodo.18162472).
