# Supplementary material for: PCSK9 Inhibitors Reduce Oxidative Stress Biomarkers in Heterozygous Familial Hypercholesterolemia
Source: J Cell Mol Med. 2026 May 24;30(10):e71206. doi: 10.1111/jcmm.71206 (PMC13239749; doi:10.1111/jcmm.71206)

**Supplementary Figure 1.** The heatmap Spearman’s rank correlation with age for the study groups:
**(A)** patients with HeFH before treatment with PCSK9 inhibitors, **(B)** HeFH patients after treatment with PCSK9 inhibitors, and **(C)** the control group. Blue squares indicate significant positive correlations
(r >0.5, *P* <0.05), white squares indicate non-significant correlations (*P* >0.05) and red squares indicate significant negative correlations (r <-0.5, *P* <0.05).


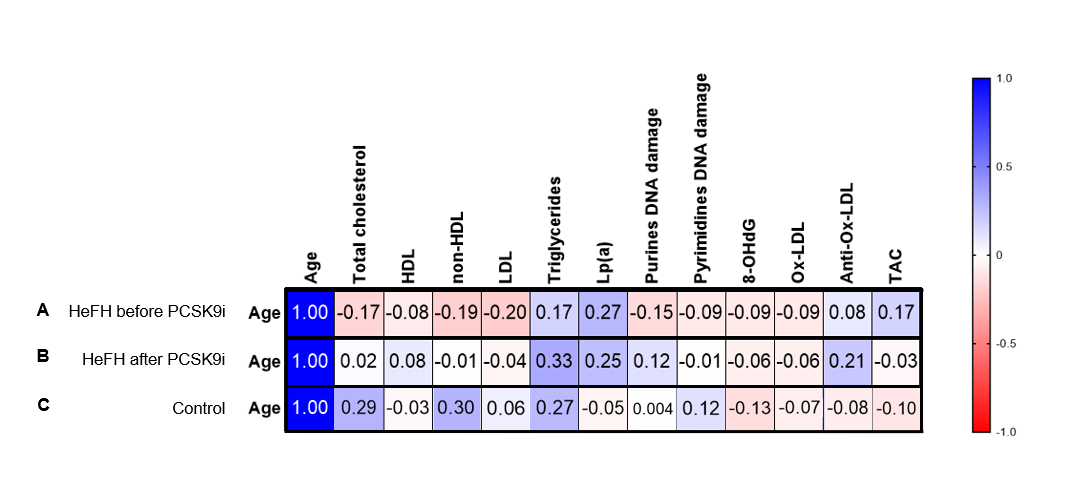

Supplement: Supplementary file 1 — Figure S1: The heatmap Spearman's rank correlation with age for the study groups: (A) patients with HeFH before treatment with PCSK9 inhibitors, (B) HeFH patients after treatment with PCSK9 inhibitors, and (C) the control group. Blue squares indicate significant positive correlations (r > 0.5, p < 0.05), white squares indicate non‐significant correlations (p > 0.05) and red squares indicate significant negative correlations (r < −0.5, p < 0.05). [file JCMM-30-e71206-s001.docx]
