# Supplementary material for: PCSK9 Inhibitors Reduce Oxidative Stress Biomarkers in Heterozygous Familial Hypercholesterolemia
Source: J Cell Mol Med. 2026 May 24;30(10):e71206. doi: 10.1111/jcmm.71206 (PMC13239749; doi:10.1111/jcmm.71206)
Supplement: Supplementary file 2 — Table S1: Serum levels of lipid parameters in controls and HeFH patients before and after treatment with PCSK9 inhibitors (Supporting Information). [file JCMM-30-e71206-s003.docx]

**Supplementary Table 1**. Serum levels of lipid parameters in controls and HeFH patients before and after treatment with PCSK9 inhibitors (Supplementary material).

| **Lipid parameters median (IQR)** | **Controls,**  ***N* = 33** | **Patients with HeFH, before PCSK9i treatment, *N* = 40** | **Patients with HeFH, after PCSK9i treatment, *N* = 40** | ***P value***  ***HeFH vs. HeFH on PCSK9i*** | ***% reduction or increase**** |
| --- | --- | --- | --- | --- | --- |
| Total cholesterol | 197 (173;221.5) | 241 (180.3; 275.8) | 126 (108.3;173) | **<.0001** | **47.2%** |
| HDL-C | 66.1 (51.4; 74.1) | 54.35 (46.3; 63.8) | 58.6 (50.4; 70) | **<.01** | **7.8%*** |
| Non-HDL | 131 (116.5; 150.5) | 183.5 (123.3; 215.3) | 76 (49; 109) | **<.0001** | **58.6%** |
| LDL-C | 102 (92; 112) | 172 (117; 196.5) | 50 (34.5; 79.2) | **<.0001** | **70.9%** |
| Triglycerides | 107 (77.5; 163) | 119 (91.5; 172) | 94 (72; 132) | **<.01** | **21%** |
| Lp(a) | 11.8 (3.9; 44) | 102.1 (16.75; 264.7) | 87.1 (9.4; 189.5) | **<.0001** | **14.7%** |
