# Supplementary material for: PCSK9 Inhibitors Reduce Oxidative Stress Biomarkers in Heterozygous Familial Hypercholesterolemia
Source: J Cell Mol Med. 2026 May 24;30(10):e71206. doi: 10.1111/jcmm.71206 (PMC13239749; doi:10.1111/jcmm.71206)
Supplement: Supplementary file 3 — Table S2: Multivariable linear regression model evaluating the effect of familial hypercholesterolemia (HeFH) on oxidative DNA damage with age included as a covariate. [file JCMM-30-e71206-s002.docx]

**Supplementary Table 2.** Multivariable linear regression model evaluating the effect of familial hypercholesterolemia (HeFH) on oxidative DNA damage with age included as a covariate.

| **Predictor** | **β (Estimate)** | **Standard error (β)** | **95% CI** | ***t value*** | ***P value*** |
| --- | --- | --- | --- | --- | --- |
| Age | 0.1222 | 0.0823 | -0.04193 to 10.56 | 1.485 | 0.1421 |
| HeFH | 18.91 | 2.184 | 14.55 to 23.26 | 8.656 | **<.00001** |
